# Clinical Innovation Poster Abstracts

**DOI:** 10.1080/24740527.2021.1914216

**Published:** 2021-04-22

**Introduction/Aim**: When simple strategies including numbing cream (NC) are ineffective for pediatric procedural pain-management, nurse-administered nitrous oxide (NANO) is safe and effective. The Pediatric Inpatient Department (PIPD) at Jim Pattison Children’s Hospital launched NANO for children over 1 year in September 2019. This study aims to identify barriers to use and determine satisfaction with NC and NANO for simple procedures.

**Methods**: In spring 2020, a 44-item anonymous survey was distributed to 398 health professionals caring for children admitted to PIPD. For inter-group variance analysis, responses were sorted by nurses, physicians, and others. We completed descriptive analysis for all items including coded open text responses regarding barriers.

**Results**: Seventy-one individuals out of 398 participated. For NC, 83.1% agreed NC is an essential pain-management option for needle pokes, 73.2% were confident to access/provide it, and 24% identified barriers (availability, time, distress). For NANO, 81.7% agreed NANO is an essential pain-management option for minor procedures, 50.7% were confident to access/provide it, and 32% identified barriers (education). Those involved in the care of a patient receiving NC or NANO in the past 6-months were satisfied with effectiveness, 83% and 85.7%, respectively.

**Discussion/Conclusions**: NC and NANO are essential procedural pain-management strategies in PIPD. A focus group with staff, patients and caregivers to identify strategies to overcome barriers to implementation of NC may inform further improvements. To address staff needs for further education, we suggest additional opportunities for NANO education be provided through existing channels such as nursing education days and pediatric grand rounds.

## References

[1] Workplace Safety and Insurance Board (2013) By the numbers: 2013 WSIB statistical report. Schedule 1 and Schedule 2. Retrievable from:http://www.wsibstatistics.ca/WSIB-StatisticalReport_S1.pdf and www.wsibstatistics.ca/WSIB-StatisticalReport_S2.pdf

[2] Ammendolia, C., Cassidy, D., Steenstra, I., Soklaridis, S., Boyle, E., Eng., S. … Côté, P. (2009). Designing a workplace return-to-work program for occupational low back pain: An intervention mapping approach. BMC Musculoskeletal Disorders, 10 (65). Retrievable from: *www.biomedcentral.com/1471-2474/10/65* 19508728 10.1186/1471-2474-10-65 PMC2700788

[3] Institute for Health Metrics and Evaluation (n.d.). Canada. Retrieved from healthdata.org

[4] Borgundvaag, B., McLeod, S., Khuu, W., Varner, C., Tadrous, M., & Gomes, T. (2018). Opioid prescribing and adverse events in opioid-naive patients treated by emergency physicians versus family physicians: a population-based cohort study. CMAJ open, 6(1), E110. 10.9778/cmajo.20170151 PMC5878958 29506986

[5] Busse, J. W., Mahmood, H., Maqbool, B., Maqbool, A., Zahran, A., Alwosaibai, A., … Buckley, D. N. (2015). Characteristics of patients receiving long-term opioid therapy for chronic noncancer pain: A cross-sectional survey of patients attending the Pain Management Centre at Hamilton General Hospital, Hamilton, Ontario. CMAJ Open, 3(3),E324–E330 10.9778/cmajo.20140126 PMC4593412 26442231

[6] Busse, J. (2017). The 2017 Canadian guideline for opioids for chronic non-cancer pain. Retrieved from: http://nationalpaincentre.mcmaster.ca/documents/Opioid%20GL%20for%20CMAJ_01may2017.pdf. 10.1503/cmaj.733313 PMC5628038 28970263

[7] Qaseem A, Wilt TJ, McLean RM, Forciea MA, for the Clinical Guidelines Committee of the American College of Physicians. (2017). Noninvasive treatments for acute, subacute, and chronic low back pain: a clinical practice guideline from the American College of Physicians. *Annals of Internal Medicine*, *166*, p. 514–530. 28192789 10.7326/M16-2367

