# Supplementary material for: Clinical Innovation Poster Abstracts
Source: Can J Pain. 2021 Apr 22;5(2):A34–47. doi: 10.1080/24740527.2021.1914216 (PMC8210873; doi:10.1080/24740527.2021.1914216)
Supplement: Related texts

# Abstract: Efficacy of Memantine for Phantom Limb Pain

**Authors:** Christian Roehmer, Samir Khan, Kaivalya Deshpande

**Affiliations:** a Vanderbilt University Medical Center, Nashville, TN, USA; b University of Pittsburgh Medical Center, Pittsburgh, PA, USA

## Abstract

**Patient**: An 18-year-old male with no significant past medical history presented with phantom limb pain secondary to a recent right-sided trans-tibial amputation.

**Case Description**: Due to complications from a motorcycle collision, the patient underwent a traumatic trans-tibial amputation and T4-T9 posterior spinal fusion (PSF). He was admitted to inpatient rehabilitation for pain control and mobility needs. The patient initially reported persistent 6/10 mid-back pain and 8/10 right-sided phantom limb pain, despite a multimodal analgesic regimen consisting of acetaminophen, gabapentin, oxycodone, heat therapy, and desensitization therapy.

**Assessment/Results**: The patient was started on a trial of memantine 5mg BID and found significant relief one day after initiation. He denied all phantom limb pain for the remainder of his two-week inpatient rehab stay. The patient’s total oxycodone requirements were gradually weaned; however, he did still require intermittent spot doses for his mid-back pain. He did not report any immediate side effects related to memantine.

**Discussion**: Memantine is an NMDA receptor antagonist that may help in limiting neuronal excitation and abnormal sensory pain manifestations. Due to its prolonged tolerability, low side-effect profile, and relatively rapid onset, its popularity as an off-label adjunct in chronic pain treatment has grown. Literature regarding memantine for phantom limb pain is limited.

**Conclusion**: Memantine may be an effective modality for treating phantom limb pain and limiting the need for higher-risk alternative analgesics during hospitalizations. Further studies are needed to determine the role of memantine in the treatment of neuropathic pain syndromes.

# Abstract: I feel your pain: Emotional regulation as a goal of CRPS therapy

**Authors:** Derek Van Dalen

**Affiliations:** Providence University and Theological Seminary, Counselling Psychology, Otterburne, MB, Canada

## Abstract

The etiology of CRPS suggests that physiological injury can influence maladaptive neurophysiological, psychological, and psychosocial outcomes. Ekman (2018) suggests an integrative and comprehensive understanding of emotions. Spiritual practices can provide adaptive emotional outcomes (Johnson, 2019). van Reekum and Johnstone (2018) propose that emotion regulation changes the underlying emotion. This suggests that emotional regulation can be a treatment goal for CRPS. The physiological and neurophysiological experience of CRPS, which includes dysfunction of the somatic and autonomic nervous systems, and maladaptation of the sympathetic and central nervous systems, can lead to increased feelings of anxiety and depression while also increasing psychological rigidity. The experience of CRPS diagnosis, as one of exclusion, can also negatively contribute to psychological well-being. The reduction of intrapsychic resources available for relationships and the unrecognized psychosocial needs of the informal support network, can increase social isolation. Experiential and behavioural based interventions encouraging emotion regulation can provide a pathway for treatment for patients with CRPS through endogenous generation of positive emotions and increasing cognitive-emotional flexibility to enhance well-being. Spirituality can be supportive of emotional regulation. A spiritually integrative approach can lead to the development of increased social support with others and enhanced relationship with the divine. As the experience of pain is shared in these sacred relationships, emotional regulation can become a collaborative, relationship enhancing experience. In this way the isolation arising from a diagnosis of CRPS can lead to strengthening of social support and provide meaning.

# Abstract: From Pain Worrier to Pain Warrior: A single psychoeducational session for the management of pain catastrophizing in pediatric pre-surgical candidates.

**Authors:** Fotini Zachariades, Christine Lamontagne, Deborah Mervitz, Brenda Martelli, Danijela Maras, Audrey Prayal-Brown

**Affiliations:** a Children’s Hospital of Eastern Ontario, Chronic Pain Service, Ottawa, ON, Canada; b Children’s Hospital of eastern Ontario, Department of Anesthesiology and Pain Medicine, Ottawa, ON, Canada; c Acute Pain Services, Department of Anesthesiology and Pain Medicine, Children’s Hospital of Ontario, Ottawa, ON, Canada; d Children’s Hospital of Eastern Ontario, Mental Health PSU, Ottawa, ON, Canada; e Anesthesia Resident, The Ottawa Hospital, Department of Anesthesiology and Pain Medicine, Ottawa, ON, Canada

## Abstract

Pain catastrophizing (PC) refers to a tendency to magnify/exaggerate the threat value of pain sensations, and has not only been associated with greater post-surgical pain intensity and opioid consumption, but also with the evolution of acute to chronic pain. PC has also been linked with greater physical/psychosocial disability and poorer response to multidisciplinary pain treatment. Similarly, parental pain catastrophizing has been found to negatively impact post-surgical pain in children and predict the level of child physical functioning in the acute recovery phase following surgery. Treating PC in parents and youth prior to surgery may facilitate improved surgical recovery, underscoring the need for screening and pre-surgical PC education and treatment to optimize surgical outcomes. Therefore, our aim is to pilot a single session psychoeducational workshop on PC for parents and youth at CHEO, ages 12-18, who are pre-surgical candidates. Parents and youth complete validated questionnaires assessing PC, mood, and anxiety pre-workshop, post-workshop, and two weeks post-surgery. Parents and youth also complete a satisfaction survey following workshop participation. This workshop was piloted in-person prior to the COVID-19 pandemic, and will be adapted for virtual implementation. Workshop content addresses pain neuroscience, PC, and adaptive coping strategies for managing pain and PC drawn from cognitive-behavioural therapy, acceptance and commitment therapy, and dialectical behaviour therapy approaches.

# Abstract: Virtual Reality in Pain Rehabilitation: Adoption, feasibility and acceptability of an innovative treatment for Canadian youth with persistent pain

**Authors:** Giulia Mesaroli, Anne Ayling Campos, Christine Castellarin-Harris, Sara Klein, Laura Simons, Luke Wilson, Jennifer Stinson

**Affiliations:** a The Hospital for Sick Children, Departments of Rehabilitation and Child Health Evaluative Sciences, and the University of Toronto, Department of Physical Therapy and Institute of Health Policy, Management and Evaluation, Toronto, ON, Canada; b The Hospital for Sick Children, Department of Rehabilitation, Toronto, ON, Canada; c Department of Anesthesiology, Perioperative and Pain Medicine, Palo Alto, CA, USA; d Mighty Immersion, Inc., New York, NY, USA; e The Hospital for Sick Children, Child Health Evaluative Sciences and the University of Toronto, Lawrence S. Bloomberg Faculty of Nursing, Toronto, ON, Canada

## Abstract

**Purpose**: Chronic pain affects the lives of 1 in 5 Canadian children and adolescents. Gold standard treatment is a ‘3-P’ approach combining pharmacology, psychological therapy and physiotherapy. Physiotherapy treatments, especially exercise, aim to reduce a child’s fear of movement and physical disability. There is an urgent need to find new and innovative ways to engage children in age-appropriate, play-based exercise to optimize physical function.

**Methods**: SickKids Hospital partnered with the Stanford Chariot Program to adopt their innovative Virtual Reality (VR) technology Fruity Feet. As part of a quality improvement project, eligible children at SickKids’ chronic pain clinic were invited to participate in VR during their physiotherapy treatment. Children completed questionnaires to report their pain intensity, level of immersion and satisfaction as well as engage in a 15-min interview to explore their experience using VR. Physiotherapists (PTs) rated the feasibility, acceptability and safety of VR.

**Results**: Eight children and adolescents (10-17 years) engaged in VR at SickKids for a total of 15 sessions. Two children experienced mild motion sickness and there were minor technical issues during 2 sessions which were subsequently resolved. Eighty-five percent of PTs rated VR as ‘helpful’ or ‘very helpful’ in conducting the therapy session and 100% were overall ‘satisfied or ‘very satisfied’. Seventy-five percent of children would ‘very much’ like to use VR in physiotherapy again.

**Conclusions**: Adopting the VR program Fruity Feet at a Canadian pediatric pain clinic is feasible and acceptable to PTs and patients. Further research is needed to understand the effects of this treatment modality on pain and function.

# Abstract: An interdisciplinary DBT/ACT group for the management of pediatric pain

**Authors:** Fotini Zachariades, Diana Sullivan Compton, Kim Prud’Homme

**Affiliations:** Children’s Hospital of Eastern Ontario, Chronic Pain Service, Ottawa, Ontario, Canada

## Abstract

Research suggests that mental health interventions in pediatric pain may focus primarily on pain-related cognitions, thereby inadequately addressing mental health comorbidities and being less effective in interrupting a potential trajectory of mental health and chronic pain from persisting on a longer-term basis. There is therefore increased need to address mental health comorbidities in pediatric pain to optimize outcomes. Given existing gaps in clinical services to address mental health concerns comorbid with chronic pain, an integrated DBT/ACT group was developed and implemented by the Chronic Pain Service (CPS) at CHEO. Prior to the COVID-19 pandemic this group was offered separately to youth involved with the CPS and their parents. Based on positive feedback for these groups, the youth group was adapted and implemented using a virtual format during the pandemic. Group content draws from Dialectical Behaviour and Acceptance and Commitment therapy approaches, and focuses on various coping skills from these approaches to be applied to the management of both emotions and pain. Topics related to mind-body connection, pain neuroscience, pain gate, pacing, functional/physical goals, and mindfulness are incorporated. The in-person group was co-facilitated by Psychology and Occupational Therapy, and the interdisciplinary nature of the virtual group was further expanded to include Physiotherapy. Novel aspects of this group therapy include the combined DBT/ACT approach, application of this towards pediatric pain management, involvement of Physiotherapy, and the virtual delivery of this group therapy. Future directions may involve application to other relevant patient populations and piloting the parent group in virtual format.

# Abstract: Overcoming Pain as a Barrier to Employment: Health2Work program integrates chiropractic care and social services in Waterloo Region

**Authors:** Jennifer V. Nash, Jessica Parish, Marg Harrington, Amy Brown, Peter Phillips, Pamela Mank

**Affiliations:** a McMaster University, Department of Anesthesia, Hamilton, Ontario, Canada; b Ontario Chiropractic Association, Stakeholder Relations, Health Policy & Program Development, Toronto, Ontario, Canada; d Coronation Chiropractic and Massage, Cambridge, Ontario, Canada

## Abstract

This poster provides an overview and early results from the Health2Work (H2W) program, which addresses pain as a barrier to employment by providing musculoskeletal (MSK) care to Ontario Works (OW) recipients in Waterloo Region.

Acute and chronic MSK conditions can have a significant impact on quality of life and the ability to pursue and maintain employment.^1,2^ Back, neck, and shoulder pain are leading causes of disability in Canada^3^ and are among the most common reasons for prescribing opioids.^4,5^ Clinical practice guidelines emphasize non-pharmacological management of pain as the first line approach for chronic non-cancer pain including MSK conditions.^6,7^ However, cost presents a significant barrier for those without public or private insurance. Furthermore, OW recipients currently do not receive job-related pain or functional ability assessment by a healthcare professional as part of their employment readiness planning.

Health2Work addresses these gaps by providing assessment, diagnosis and MSK care including education, exercise and manual therapy to social services recipients. Early evaluation results have demonstrated: 1) a significant demand for the program; 2) participants are motivated by the H2W program because they are seeking pain relief, want to receive care for acute and chronic conditions, and want to be ready for employment; 3) positive interactions with case workers/employment facilitators and chiropractors facilitate continued participation in the program; and 4) H2W has been successful in helping participants return to work, and enter re-training programs.

Health2Work is a partnership between the Region of Waterloo, Langs Community Health Centre in Cambridge,and the Ontario Chiropractic Association.

## References

[1] Workplace Safety and Insurance Board (2013) By the numbers: 2013 WSIB statistical report. Schedule 1 and Schedule 2. Retrievable from:http://www.wsibstatistics.ca/WSIB-StatisticalReport_S1.pdf and www.wsibstatistics.ca/WSIB-StatisticalReport_S2.pdf

[2] Ammendolia, C., Cassidy, D., Steenstra, I., Soklaridis, S., Boyle, E., Eng., S. … Côté, P. (2009). Designing a workplace return-to-work program for occupational low back pain: An intervention mapping approach. BMC Musculoskeletal Disorders, 10 (65). Retrievable from: *www.biomedcentral.com/1471-2474/10/65*1950872810.1186/1471-2474-10-65PMC2700788

[3] Institute for Health Metrics and Evaluation (n.d.). Canada. Retrieved from healthdata.org

[4] Borgundvaag, B., McLeod, S., Khuu, W., Varner, C., Tadrous, M., & Gomes, T. (2018). Opioid prescribing and adverse events in opioid-naive patients treated by emergency physicians versus family physicians: a population-based cohort study. CMAJ open, 6(1), E110.10.9778/cmajo.20170151PMC587895829506986

[5] Busse, J. W., Mahmood, H., Maqbool, B., Maqbool, A., Zahran, A., Alwosaibai, A., … Buckley, D. N. (2015). Characteristics of patients receiving long-term opioid therapy for chronic noncancer pain: A cross-sectional survey of patients attending the Pain Management Centre at Hamilton General Hospital, Hamilton, Ontario. CMAJ Open, 3(3),E324–E33010.9778/cmajo.20140126PMC459341226442231

[6] Busse, J. (2017). The 2017 Canadian guideline for opioids for chronic non-cancer pain. Retrieved from: http://nationalpaincentre.mcmaster.ca/documents/Opioid%20GL%20for%20CMAJ_01may2017.pdf.10.1503/cmaj.733313PMC562803828970263

[7] Qaseem A, Wilt TJ, McLean RM, Forciea MA, for the Clinical Guidelines Committee of the American College of Physicians. (2017). Noninvasive treatments for acute, subacute, and chronic low back pain: a clinical practice guideline from the American College of Physicians. *Annals of Internal Medicine*, *166*, p. 514–530.2819278910.7326/M16-2367

# Abstract: Optimizing the management of pain and irritability of unknown origin in children with severe neurological impairment

**Authors:** Hal Siden, Bruce Carleton, Tammie Dewan, Liisa Holsti, Vithya Gnanakumar, Julia Orkin, Anamaria Richardson, Marie-Claude Grégoire, Christina Vadeboncoeur, Anne-Mette Hermansen, Tim Oberlander

**Affiliations:** a Children’s Hospital, University of British Columbia, Department of Pediatrics, Vancouver, British Columbia, Canada; b Alberta Children’s Hospital, University of Calgary, Department of Pediatrics, Calgary, Alberta, Canada; c Children’s Hospital, University of British Columbia, Department of Occupational Science and Occupational Therapy, Vancouver, BC, Canada; d Hospital for Sick Children, University of Toronto, Department of Pediatrics, Toronto, Ontario, Canada; e Children’s Hospital of Eastern Ontario, University of Ottawa, Department of Pediatrics, Ottawa, Ontario, Canada

## Abstract

Pain is the most common symptom reported by parents of children with severe neurological impairments (SNI), but when children can’t tell us where it hurts, parents and healthcare providers rely on interpretation of nonspecific pain-like behaviours to detect the presence of pain. These signs are challenging to evaluate and cannot always be attributed to a specific cause. Moreover, in children with complex neurological conditions, pain-like behaviours are not always due to disease or injury (nociceptive-inflammatory, or nociceptive), but instead may be due to generalized irritability of the central nervous system (nociplastic). When we cannot determine the origin of the sensation we call it *Pain and Irritability of Unknown Origin (PIUO)*.

To date, little is known about whether biochemical, physiological or structural neurological changes associated with SNI contribute to the pain experience or how to develop a systematic approach to treatment. Since we cannot often make the same assumptions and inferences about pain signals or responses as we might observe in a typically developing child, we need better tools to address pain in these children.

Our research evaluates a clinical pathway to see if a systematic approach to PIUO can lead to better outcomes. This innovative clinical pathway, evaluated through a randomized controlled trial, combines comprehensive assessments, directed testing and targeted screenings with dedicated nursing support to manage pain and irritability on a daily basis. We show how a focused intervention paired with a dedicated support system will make a difference for children and their families.

# Abstract: Bridging the gap: Development of Collaborative to improve integration of non-pharmacological treatment options for musculoskeletal pain

**Authors:** Jennifer V. Nash, Marg Harrington, Benjamin Xafflorey, Wilhelm Wojcik, Amy Brown

**Affiliations:** a McMaster University, Department of Anesthesia, Hamilton, Ontario, Canada; b Ontario Chiropractic Association, Stakeholder Relations, Health Policy & Program Development, Toronto, Ontario, Canada; c Ontario Chiropractic Association, Operations (Project Management and Privacy Office), Toronto, Ontario, Canada; d Ontario Chiropractic Association, Communications and Marketing, Toronto, Ontario, Canada; e Coronation Chiropractic and Massage, Cambridge, Ontario, Canada

Musculoskeletal (MSK) conditions are among the top causes of disability globally^1^. They impact a person’s ability to work and socialize, while impeding their physical and mental well-being. Clinical practice guidelines recommend non-pharmacological treatment as the first line of care, including education, exercise and manual therapy^2,3,4^. A systematic review found that people with neck and back pain who used chiropractic care were less likely to be prescribed opioids^5^. In addition, 83% of Ontario patients seen in the government-funded Primary Care Low Back Pain Clinics report requiring less medications after seeing an MSK expert^6^. Despite these recommendations, low back pain is the top reason for initial opioid prescription in Ontario’s family practices and emergency departments^7^.

To address this situation, the Ontario Chiropractic Association developed the Opioid and Pain Reduction Collaborative. This Collaborative will help bridge the gap in how and when to manage MSK pain with non-pharmacological treatment, including referral to and collaboration with chiropractors, physiotherapists and/or registered massage therapists as supported by best-available evidence. The Collaborative includes a clinical tool^8^, developed by the Centre for Effective Practice, to improve primary care providers’ confidence in implementing a multi-modal care plan for MSK pain. A separate, complementary toolkit^9^ helps chiropractors initiate conversations about MSK pain management options, within their scope of practice, with patients and their primary care providers. The intent is to empower health professionals to collaborate with their patients and colleagues and leverage non-pharmacological treatment.

This poster will outline the rationale for the Collaborative, its development, key components and implementation plan.

## References

[1] James SL, Abate D, Abate KH, Abay SM, Abbafati C, Abbasi N, et al. Global, regional, and national incidence, prevalence, and years lived with disability for 354 diseases and injuries for 195 countries and territories, 1990–2017: a systematic analysis for the Global Burden of Disease Study 2017. The Lancet. 2018 Nov;392(10159):1789–1858.10.1016/S0140-6736(18)32279-7PMC622775430496104

[2] Stochkendahl MJ, Kjaer P, Hartvigsen J, Kongsted A, Aaboe J, Andersen M, et al. National Clinical Guidelines for non-surgical treatment of patients with recent onset low back pain or lumbar radiculopathy. Eur Spine J. 2018 Jan;27(1):60–75.2842914210.1007/s00586-017-5099-2

[3] Wong JJ, Côté P, Sutton DA, Randhawa K, Yu H, Varatharajan S, et al. Clinical practice guidelines for the noninvasive management of low back pain: A systematic review by the Ontario Protocol for Traffic Injury Management (OPTIMa) Collaboration. Eur J Pain. 2017 Feb;21(2):201–16.2771202710.1002/ejp.931

[4] Busse JW, Craigie S, Juurlink DN, Buckley DN, Wang L, Couban RJ, et al. Guideline for opioid therapy and chronic noncancer pain. CMAJ. 2017 May 8;189(18):E659–66.2848384510.1503/cmaj.170363PMC5422149

[5] Corcoran KL, Bastian LA, Gunderson CG, Steffens C, Brackett A, Lisi AJ. Association Between Chiropractic Use and Opioid Receipt Among Patients with Spinal Pain: A Systematic Review and Meta-analysis. Pain Medicine. 2019 Sep 27;pnz219.10.1093/pm/pnz21931560777

[6] Primary Care Low Back Pain Pilot Evaluation: Final Report. Centre for Effective Practice. Toronto: Ontario. March 2017. https://hqontario.ca/Portals/0/documents/evidence/quality-standards/qs-low-back-pain-recommendations-for-adoption-en.pdf

[7] Borgundvaag B, McLeod S, Khuu W, Varner C, Tadrous M, Gomes T. Opioid prescribing and adverse events in opioid-naive patients treated by emergency physicians versus family physicians: a population-based cohort study. CMAJ Open. 2018 Mar 1;6(1):E110–7.10.9778/cmajo.20170151PMC587895829506986

[8] Manual Therapy as an Evidence-Based Referral for Musculoskeletal Pain Tool. Centre for Effective Practice. Toronto: Ontario. June 2020. https://cep.health/clinical-products/manual-therapy/

[9] Pain Reduction Toolkit. Ontario Chiropractic Association. Toronto: Ontario. September 2020. https://chiropractic.on.ca/wp-content/uploads/Pain-Reduction-Chiropractic-Dialogue-Map-1-1.pdf

# Abstract: User perceptions of mycarepath.ca, a website designed to provide self-management strategies for adolescents with chronic pain

**Authors:** Kamal Sidhu, Nicholas West, Sarah Kuhn, Rachel Ku, Ethan Ponton, Gillian Lauder

**Affiliations:** a University of British Columbia, Anesthesiology Pharmacology & Therapeutics, Vancouver, BC, Canada; b BC Children’s Hospital, Office of Pediatric Surgical Evaluation and Innovation, Vancouver, BC, Canada

## Abstract

Chronic pain is a complex biopsychosocial experience that causes significant distress and reduced quality of life in up to 6% of Canadian youth [1]. Trapped in a downwards spiral of worsening pain, sleep, school attendance, family relationships and mood, these children and adolescents require early, meaningful help to prevent further deterioration. Evidence suggests that self-management can be an effective strategy to reduce chronic pain [2].

Mycarepath.ca is a web-based resource for children and adolescents suffering with ongoing chronic pain; it aims to improve understanding of pain and to promote self-management strategies, as well as provide resources for parents and teachers [3]. Since May 2016, mycarepath.ca has been accessed by up to 80 users per day, with the majority (60%) located in Canada. It has proven to be a useful resource for the BC Children’s Hospital Complex Pain Service: mycarepath.ca is recommended to families awaiting an appointment and patients are referred back to the website during their care.

We are now in the process of upgrading this website. To guide this development, we have created an electronic survey, using REDCap [4]. The survey will be distributed to our adolescent patients and their parents to understand their use and perceptions of mycarepath.ca. Results, available in early 2021, will help our team produce a more patient- and family-centred website. It is anticipated that this information will be useful to other complex pain practitioners, developing or using a similar website, to support children and their families in their journey to recover from chronic pain.

## References

[1] van Dijk et al. Pain Res Manag. 2006;11(4):234–40.1714945610.1155/2006/835327PMC2673140

[2] Newman et al. Lancet. 2004;364(9444):1523–37.1550089910.1016/S0140-6736(04)17277-2

[3] UBC Department of Pediatrics. www.mycarepath.ca [last accessed 01-Nov–2020].

[4] Harris et al. J Biomed Inform. 2009;42(2):377–81.1892968610.1016/j.jbi.2008.08.010PMC2700030

# Abstract: A comparison of results of a pain reprocessing group intervention offered in person versus via videoconferencing: The impact of COVID-19

**Authors:** Pamela L. Holens, Martine Southall, Catherine Desorcy-Nantel, Adair Libbrecht, Jeremiah Buhler, Liana Rock

**Affiliations:** a University of Manitoba, Clinical Health Psychology, Winnipeg, Manitoba, Canada; b University of Manitoba, Psychology, Winnipeg, Manitoba, Canada

## Abstract

**Introduction**: Chronic pain is highly prevalent among military, RCMP, and veterans. In a previous study, we had found that a 10-week in-person Pain Reprocessing Therapy was effective in reducing pain and pain-related disability in this population. Due to the COVID-19 pandemic, the treatment delivery was necessarily modified to an online video-conferencing format from the previous in-person group format. The goal of this study was to evaluate whether this change in delivery format impacted the effectiveness of the intervention.

**Methods**: Participants in this study were military, RCMP, and veterans who participated in a 10-week Pain Reprocessing Therapy at an Operational Stress Injury Clinic. Analyses examined 15 individuals who completed the intervention in person to a matched 15 individuals who completed the treatment using a videoconferencing platform. Participants completed pain-related questionnaires pre- and post-treatment, as well as a treatment satisfaction questionnaire involving quantitative and qualitative questions.

**Results**: There were minimal differences in pain-related and satisfaction-related results when comparing the intervention in person participants to those who completed through videoconferencing. The only significant difference that was noted when the format was moved to videoconferencing due to the pandemic is that the dropout numbers increased slightly in the videoconference-based groups.

**Discussion**: Results of this study indicate that our Pain Reprocessing Therapy group treatment, whether offered in person or through videoconferencing, was equally efficacious and equally well received within the population studied, although dropout rates were higher in the latter format. Further research in the form of a randomized controlled trial is warranted.

# Abstract: Evaluating the role of positive and negative psychological factors at three months after back pain

**Authors:** Zakir Uddin, Arthur Woznowski-Vu, Timothy H. Wideman

**Affiliations:** a McGill University, School of Physical and Occupational Therapy, Montreal, Quebec, Canada

## Abstract

**Introduction/Aim**: The study was aimed to examine the role of baseline positive-negative psychological factors at 3 months after back pain.

**Methods**: In a longitudinal cohort, the study evaluated the (1) predictive value of negative psychological factors beyond positive psychological factors considering covariates in pain-related outcomes at 3 months, (2) effects of baseline high-low positive psychological factors in outcomes at baseline and 3-months.

**Results**: Among negative factors, pain catastrophizing was a predictor of affective interference (β = .332, t = 2.775, p < .01), physical interference (β = .256, p < .05) and disability (β = .311, p < .01) outcomes (3 out of 4 outcomes in this study); and kinesiophobia (β = .227, p < .05) was a predictor of physical interference outcome. Among positive factors, only resilience was significantly correlated with physical interference outcome (r = -.242, p<.05). However, resilience was not a significant predictor in the regression model but the negative psychological factors were significantly contributed an additional 15.4% variance beyond the other baseline factors in predicting physical interference. There were significant differences in the pain catastrophizing for high-low resilience (t=-2.240, p=.028) and high-low compassion (t = -2.829, p=.006) categories and it indicated 2 positive factors (resilience and compassion) are inversely related to the negative factor (catastrophizing).

**Discussion/Conclusions**: Negative psychological factor (i.e. pain catastrophizing) is the strongest predictor for most of the outcomes. An inverse relationship exists between positive and negative psychological factors. The study evaluates the positive-negative valence of back pain rehabilitation, and findings have important theoretical and clinical implications.

# Abstract: Treating pain in the family: A virtual psychological intervention for parent chronic pain in the context of pediatric pain care

**Authors:** Kathryn A. Birnie, Melanie Noel, Isabel Jordan, Janice Sumpton, Maria Pavlova, Sara Ahola Kohut, Kyleigh Schraeder, Serena L. Orr, Lindsay Craddock

**Affiliations:** a University of Calgary, Department Anesthesiology, Perioperative, and Pain Medicine, and Department of Community Health Sciences, Calgary, AB, Canada; b University of Calgary, Department of Psychology, Calgary, AB, Canada; c Patient Partner, Squamish, BC, Canada; d Patient Partner, London, ON, Canada; e The Hospital for Sick Children, Department of Gastroenterology, Hepatology and Nutrition; University of Toronto, Department of Psychiatry, Toronto, ON, Canada; f Department of Pediatrics, Calgary, AB, Canada; g Alberta Children’s Hospital, Pediatric Self-Management Clinic, Calgary, AB, Canada

## Abstract

**Background**: Chronic pain runs in families. Approximately 50% of children with chronic pain have a parent with chronic pain; furthermore, children of parents with chronic pain are at greater risk for pain, emotional, behavioural, and family problems. Intergenerational models have delineated possible biopsychosocial mechanisms through which parental chronic pain contributes to pediatric chronic pain. Pediatric pain interventions primarily focus on parents’ responses to their child’s pain. The lack of content addressing parents’ own pain and mental health is problematic given the high prevalence of these concerns amongst parents of children with chronic pain, their impact on parent behaviours, and child pain. Long waitlists and poor access to care are barriers to addressing parent and child chronic pain concurrently across pediatric and adult health systems.

**Clinical Innovation**: We developed a virtual group-based Acceptance and Commitment Therapy (ACT) for parents with chronic pain who have a child with chronic pain. The group comprises four 90-minute weekly sessions delivered over Zoom. The first three sessions are modelled after other brief ACT interventions for adults with chronic pain shown to improve pain, mental health, and functioning. The fourth session focuses on parenting with chronic pain. The parent group occurs alongside a five 90-minute weekly virtual group-based psychological intervention for children 10-17 years old with chronic pain. Seven parent-child dyads are participating February-March 2021. Pre- and post-group surveys and interviews will assess feasibility and preliminary intervention effectiveness. This novel parent intervention offers a new avenue in care to address pain as an intergenerational health issue.

# Abstract: Therapeutic Yoga Interventions for Chronic Pain

**Authors:** Ting Qi (Amy) Huang, Eleni G. Hapidou

**Affiliations:** a McMaster University, Faculty of Health Sciences, Hamilton, Ontario, Canada; b Hamilton Health Sciences and McMaster University, Michael G. DeGroote Pain Clinic, Hamilton, Ontario, Canada

## Abstract

The practice of Yoga integrates all aspects of the person, with biological, mental, intellectual, and spiritual connections. Yoga aligns with holistic principles of the Biopsychosocial perspective, and as such, it can be instrumental in the treatment of chronic pain. The purpose of this review is to explore the impact of Yoga interventions on chronic pain through reviewing Yoga literature and research studies. Results indicate that Yoga has beneficial impacts on participants, with documented reductions in pain (as measured by patient-reported outcomes) and increased volumes of brain structures. Comparisons and connections were also drawn between Yoga and standard therapies for chronic pain such as Cognitive Behavioral Therapy, Mindfulness Meditation, and Acceptance and Commitment Therapy. The research studies reviewed here were often limited by small sample sizes and unequal numbers of men and women. Future studies should focus on recruiting a larger number of participants to allow for increased statistical power and generalizability of results. In-depth study regarding the practice of Yoga in men is necessary to expand the knowledge of the physical and mental impact of Yoga practice on both sexes. Yoga is an ever-evolving health practice, and further research would be essential to uncover the full benefits of Yoga for patients with chronic pain.

# Abstract: A National, Innovative Pain Management Curricula for Postgraduate Medical Education and Continuing Professional Development

**Authors:** Nancy Dalgarno, Amber Hastings Truelove, Jennifer Turnnidge, Angela Coderre-Ball, Britney Lester, Shannon Hill, Jessica Baumhour, Richard van Wylick, Fran Kirby, Lisa Graves

**Affiliations:** a Queen’s University, Office of Professional Development and Educational Scholarship (OPDES), Kingston, Ontario, Canada; b Queen’s University, OPDES, Kingston, Ontario, Canada; c Association of Faculties of Medicine of Canada, St. John’s, NL, fkirby@afmc.ca; d Western Michigan University Homer Stryker M.D. School of Medicine, Kalamazoo, Michigan, U.S.A

## Abstract

**Background**: In January 2021, the Association of the Faculties of Medicine of Canada (AFMC), launched an online pain management and opioid stewardship curriculum for integration into Canada’s 17 medical school programs. To scaffold this initiative, AFMC is developing an extension of this program for postgraduate medical education (PGME) and continuing professional development (CPD).

**Methodology**: To aid in the development of the PGME program, we conducted an environmental scan learning needs and identify existing pain management resources. The environmental scan consisted of a scoping review of patient experiences with pain and opioid management, and document analyses of Canadian Pain Guidelines and Competency Frameworks, the CanMEDS key and enabling competencies, the College of Family Physicians of Canada’s 105 Priority Topics, and PGME pain-related curricula from Canada’s 17 medical schools. We also surveyed key stakeholders in PGME and CPD offices and interviewed stakeholders from partner associations.

**Results**: Current PGME programs have been praised for their value, interactions with others, and up-to-date evidence. Future PGME programs should ensure that curricula focus on patient-centered approach to care, the stigma and bias around opioid use and prescribing, evidence-based guidelines, and developing competencies based on CanMEDS and CFPC skill dimensions.

**Next Steps**: Through a collaborative approach, AFMC is currently working with experts in the field, and patient and family advocates to begin identifying the key topics and learning outcomes for the PGME and CPD curricula.

# Abstract: Introducing remote self-management strategies in a chiropractic teaching clinic in Spain during the COVID–19 lockdown: An observational study

**Authors:** Carlos Gevers-Montoro, Zoha Deldar, Andrea Furlan, Eric A. Lazar, Erfan Ghalibaf, Ali Khatibi, Arantxa Ortega de Mues

**Affiliations:** a RCU María Cristina, Chiropractic, San Lorenzo de El Escorial, Madrid, Spain and Université du Québec à Trois-Rivières, Anatomy, Trois-Rivières, Québec, Canada; b Université du Québec à Trois-Rivières, Anatomy, Trois-Rivières, Québec, Canada; c University of Toronto, KITE, Toronto Rehabilitation Institute, University Health Network, Institute for Work & Health, Toronto, Canada; d RCU María Cristina, Chiropractic, San Lorenzo de El Escorial, Madrid, Spain; e Institute for Cognitive Science Studies (ICSS), Tehran, Iran; f University of Birmingham, Centre of Precision Rehabilitation for Spinal Pain, Birmingham, UK

## Abstract

**Introduction/Aim**: In March 2020, a state-wide confinement was declared in Spain. Remotely supported activities were prioritized as an alternative to in-person interactions. Telehealth prescription of self-management strategies (SMS) previously showed to be successful at reducing pain, anxiety and catastrophizing. The present study aimed to investigate the effects of a novel telehealth program based on SMS on pain and associated psychological factors at the onset of the COVID–19 pandemic in Spain.

**Methods**: A cohort of 208 patients from the Madrid College of Chiropractic student clinic was recruited to participate in the study. Patients received telehealth consultations and a personalized video demonstrating SMS tailored for their current complaint, mainly physical exercise. Patients applied these strategies daily for a minimum of 14 and a maximum of 28 days, while rating their pain intensity, motivation and adherence. Online questionnaires were used to assess catastrophizing, kinesiophobia and generalized anxiety.

**Results**: A total of 168 patients completed the first 2 weeks of the program; 40 reached the 4-week follow-up. Pain intensity was significantly reduced after 2 days of implementation of the SMS. Catastrophizing, kinesiophobia and anxiety significantly decreased after 2 weeks, compared to baseline. All variables excluding kinesiophobia, remained lower at the 4-week follow-up.

**Discussion/Conclusions**: After only 2 weeks of implementation, a novel telehealth program based on SMS was associated with a reduction in pain intensity, pain catastrophizing and anxiety during the COVID–19 lockdown in Spain. In the context of COVID–19, remote approaches may be considered for minimizing pain and associated psychological factors.

# Abstract: Extension for Community Healthcare Outcomes (ECHO) Chronic Pain and Opioid Stewardship in Northern Ontario: Thematic Analysis of 40 Case Discussions

**Authors:** Adrian Grebowitz, Ines Almeida, Rosemee Cantave, Donna Garstin, Andrew Koscielniak, Bryan MacLeod, Patricia A. Poulin

**Affiliations:** a Ontario School of Medicine at Lakehead University, Thunder Bay, ON, Canada; b Social and Organizational Psychology; ISCTE-IUL, Portugal; Graduate Research Assistant, The Ottawa Hospital Research Institute; c Universite Notre-Dame d’Haiti, Research Coordinator, The Ottawa Hospital Research Institute; Department of Anesthesiology and Pain Medicine, Ottawa, Ontario, Canada; d St. Joseph’s Care Group, Department of Community Mental Health, Thunder Bay, Ontario, Canada; e St. Joseph’s Care Group, Chronic Pain Management Program, Thunder Bay Ontario, Canada; f The Ottawa Hospital Research Institute; Department of Anesthesiology and Pain Medicine, University of Ottawa, Ottawa, Ontario, Canada

## Abstract

Project Extension for Community Healthcare Outcomes Chronic Pain and Opioid Stewardship is an evidence-based educational program connecting primary care providers with an interprofessional team by videoconference to learn about managing chronic non-cancer pain. Each session includes a didactic presentation and a de-identified patient case. This evaluation identified common themes in cases to guide the development of future sessions. Method: We reviewed 40 consecutive cases presented by family physicians (35%) and nurse practitioners (30%). Two independent reviewers extracted patient demographic and medical characteristics from the case presentation form and coded key learning points documented on the case recommendation form using the constant comparative method. Results: Patients (52.5% males) were on average 54.6 years old (SD 18.1). The most common pain locations were the back (27.5%), extremities (22.5%), and head/neck (17.5%), with the majority of patients reporting pain in more than three locations (77.5%). Fifty percent of patients were using short-acting opioids and 30% were using long-acting opioids. Substance use was prominent including alcohol (32.5%), nicotine (32.5%), cannabis (27.5%) and illicit opioids (22.5%). Thematic analysis of the case recommendations revealed key learning points within six major themes: 1. Assessment and diagnosis 2. Pharmacological pain and symptom management (including opioid prescription and management) 3. Non-pharmacological pain and symptom management 4. Interventional management 5. Attention to biopsychosocial factors 6. Referral to other health care providers. Conclusion: We identified key knowledge gaps commonly discussed during ECHO sessions. We are building a library of resources targeting these areas of practice for our Project ECHO participants.

# Abstract: Chest binding in Ireland. A complex pain patient and the importance of the bio-pyscho-social model in management

**Authors:** Miriam Molloy, Dominic Harmon

**Affiliations:** University Hospital Limerick, Department of Pain Medicine, Limerick, Co. Limerick, Ireland

## Abstract

Binding is the practice of compressing breast tissue to give an appearance of a flat chest. This is important for many patients including transgender, non-binary and masculine-presenting assigned females at birth.

We convey a case of a 17-year-old masculine-presenting assigned female at birth who presented to a pain clinic appointment with new complaints of bilateral chest wall pain. There is an additional layer of complexity due to a background history of Ehlers-Danlos Syndrome (EDS) Type III and Pain Amplification Syndrome (PAS), Insomnia and Anxiety. On clinical examination tenderness at the costochondral region, lateral ribs and costovertebral joints bilaterally was appreciated. The majority of the pain has now resolved after changes in binding practice however patients with EDS may take prolonged time periods to recover from injury and clinical examination assessing progress may be obscured secondary to PAS.

This case demonstrates binding can cause pain and may have long term health implications. Pain may have been amplified due to EDS and PAS. A key aspect was the non-judgmental, open communication style by the pain specialist enabling the patient to interact in honest dialogue with their doctor. The decision to augment binding methods was made by the patient with support from the pain MDT ultimately alleviating symptoms whilst respecting autonomy. This case demonstrated a delicate balance of prioritising health needs, treating pain whilst simultaneously respecting gender expression and treating the patient holistically.

# Abstract: Graduation day: creation of a combined adult/pediatric pain clinic to address wait time

**Authors:** Christine Lamontagne

**Affiliations:** University of Ottawa, Anesthesiology and Pain Medicine, Ottawa, Ontario, Canada

## Abstract

Chronic pain affects one in five Canadian children and causes severe disability in about 5-8% of youth. When pain affects a child’s quality of life, its management requires a multidisciplinary approach that addresses its physical, pharmacological and psychosocial dimensions. The Chronic Pain Service at CHEO offers this multipronged pain treatment and follows between 150-200 patients yearly. Unfortunately, a significant proportion of adolescents continue to have chronic pain into adulthood and require ongoing health services for pain management. Many of these patients are transitioned to the care of their primary healthcare providers, community physiotherapists or community psychologists but about 10% -20% still require specialized care provided by the multidisciplinary adult pain clinic. Historically, young adult patients were facing long wait times (2-5 years) to access the adult pain clinic at the Ottawa General Hospital. In 2014, a combined paediatric and adult pain clinic was created in order to facilitate transition to adult pain care. This multidisciplinary transition clinic now runs every 3-4 months and allows members of the adult pain team to meet with the youth, the parents, and the involved pediatric pain health care providers. The purpose of this combined effort is to handover care to the adult pain team by providing a very comprehensive history and treatment to date, establish an ongoing need assessment, explain the adult pain care model, answer questions from the patient and family in a collaborative atmosphere, and align their expectations with the current adult service delivery model. Since the establishment of this initiative, young adults who turn 18 and require ongoing pain care have had a seamless transition to the multidisciplinary adult pain clinic at the Ottawa General Hospital, with a shorter wait time (0-3 months) and better preparation to learn how to advocate and manage in an autonomous fashion.

# Abstract: Lumbar Spinal Epidural Lipomatosis Secondary to Obesity

**Authors:** Christian Roehmer, Samir Khan, Kaivalya Deshpande

**Affiliations:** a Vanderbilt University Medical Center, Nashville, TN; b University of Pittsburgh Medical Center, Pittsburgh, PA

## Abstract

**Patient**: A 73-year-old male with a past medical history of hypertension, type II diabetes, and morbid obesity presented with chronic low back pain.

**Case Description**: The patient presented to the spine clinic exhibiting chronic lumbar back pain of several years’ duration. He endorsed non-radiating pain that was 5/10 in severity and worse on the right. The pain worsened with walking and standing and improved with sitting. He had tried physical therapy without much benefit and was taking NSAIDs as needed. His physical exam was significant for obesity and limited lumbar flexion and extension; dermatomes and myotomes were intact.

**Assessment/Results**: A lumbar MRI was performed that was significant for diffuse degenerative changes and the “Y” sign of epidural lipomatosis. The patient was recommended to lose weight as an initial conservative treatment.

**Discussion**: Epidural lipomatosis is a rare condition that typically presents insidiously. It has a higher prevalence in the overweight population and is caused by excess deposition of adipose tissue in the spinal canal secondary to exogenous steroids, Cushing’s syndrome, obesity, or idiopathic causes. Conservative treatment involves addressing any possible underlying cause before more invasive measures are taken.

**Conclusion**: Conservative treatment with weight loss may be may be a reasonable consideration for initial treatment of mild lumbar back pain in obese patients with epidural lipomatosis. Further studies should be done measuring the effectiveness of weight loss as a treatment for epidural lipomatosis.

# Abstract: Designing and Developing a Comprehensive Pain Neuroscience Education Package for Patients with Chronic Pain

**Authors:** Zahra Ofoghi, Pegah Kiaei, Diane Gromala

**Affiliations:** a Mitacs Elevate Post-Doctoral Fellow at Pain Studies Lab, School of Interactive Arts & Technology, Simon Fraser University, Surrey, British Columbia, Canada; b Pain Studies Lab, School of Interactive Arts & Technology, Simon Fraser University, Surrey, British Columbia, Canada; c Distinguished Professor, School of Interactive Arts & Technology, Simon Fraser University, Surrey, British Columbia, Canada

## Abstract

Pain Neuroscience Education (PNE) has been shown to be effective in reducing the burden of pain in patients with chronic pain. These PNE sessions are designed to explain topics such as the neuroscience of chronic pain development; Learning about these issues has demonstrated to be effective in alleviation of dysfunctionality and pain catastrophizing in patients with chronic pain. However, PNE sessions are often limited as most are organized as in-person workshops led by clinicians and experts in the field, and they are not adjusted based on the learning ability of each patient. Thus, we designed a Comprehensive Patient-centered Pain Education (CoPPE) package that has three main parts: 1) An in-person orientation session; 2) Seven virtual and interactive educational interventions (30-minutes each); 3) A closing in-person session. The virtual educational interventions include: completing a pain diary, watching a short 5-minute video, and one easy-to-perform activity to promote lifestyle changes. For the first version, video topics include: the Neuroscience of Pain, the Biopsychosocial Model of Pain, the Biological Aspects of Pain, Psychological Aspects of Pain, Social Aspects of Pain, the Neuroscience of Pain Management Interventions, and Novel Pain Management Interventions. This version also provides patients with evidence-based information about positive lifestyle changes through each intervention.

After creating the CoPPE package, we will run pilot a proof-of-concept study. We expect that by completing the CoPPE package, patients’ pain intensity may be reduced, and pain disability measures and pain catastrophizing may be improved. After assessing and refining CoPPE, we expect that patients will experience a better quality of life by completing this educational package.

**Conflict of interest** This research was supported by a Mitacs Elevate award and, through this award, by Easa Therapeutics. Dr. Gromala acknowledges a conflict of interest as the owner of Easa Therapeutics.
